# *Mycobacterium massiliense* Induces Macrophage Extracellular Traps with Facilitating Bacterial Growth

**DOI:** 10.1371/journal.pone.0155685

**Published:** 2016-05-18

**Authors:** Sungmo Je, Hailian Quan, Yina Yoon, Yirang Na, Bum-Joon Kim, Seung Hyeok Seok

**Affiliations:** Department of Microbiology and Immunology, and Institute of Endemic Disease, Seoul National University College of Medicine, Seoul, 110–799, Republic of Korea; Hopital Raymond Poincare - Universite Versailles St. Quentin, FRANCE

## Abstract

Human neutrophils have been known to release neutrophil extracellular traps (NETs), antimicrobial DNA structures capable of capturing and killing microbes. Recently, a similar phenomenon has been reported in macrophages infected with various pathogens. However, a role for macrophages extracellular traps (METs) in host defense responses against *Mycobacterium massiliense* (*M*. *mass*) has yet to be described. In this study, we show that *M*. *mass*, a rapid growing mycobacterium (RGM), also induces the release of METs from PMA-differentiated THP-1 cells. Intriguingly, this process is not dependent on NADPH oxidase activity, which regulates NET formation. Instead, *M*. *mass*-induced MET formation partially depends on calcium influx and requires phagocytosis of high bacterial load. The METs consist of a DNA backbone embedded with microbicidal proteins such as histone, MPO and elastase. Released METs entrap *M*. *mass* and prevent their dissemination, but do not have bactericidal activity. Instead, they result in enhanced bacterial growth. In this regard, METs were considered to provide interaction of *M*. *mass* with cells and an environment for bacterial aggregation, which may facilitate mycobacterial survival and growth. In conclusion, our results demonstrate METs as an innate defense response against *M*. *mass* infection, and suggest that extracellular traps play a multifaceted role in the interplay between host and bacteria.

## Introduction

The *Mycobacterium abscessus* (*M*. *abscessus*) complex is a group of rapidly growing nontuberculous mycobacteria (NTM) that cause inflammatory disease ranging from localized abscess to disseminated infection in patients with immune deficiency such as cystic fibrosis or immunocompromised patients [1, 2, 3]. The *M*. *abscessus* complex comprises of three genetically related species: *M*. *abscessus* (sensu stricto), *M*. *massiliense*, and *M*. *bolletii* [4]. *M*. *abscessus* and *M*. *massiliense* (*M*. *mass*) are often isolated from patients with respiratory disease, and are known to be hard to treat and take longer to cure because they often acquire resistance to multiple antibiotics [5].

Various infectious agents such as viruses, bacteria and fungi have been known to cause release of extracellular DNA structures from neutrophils, macrophages, eosinophils, and mast cells [6, 7, 8]. The structure, termed neutrophil extracellular traps (NETs) as they were first found in neutrophils, is known to entrap and expose microorgnisms to clearance by other immune cells [6, 9]. Extracellular traps comprise of DNA, histones, and antimicrobial enzymes that have bactericidal effects [6, 10]. Activated neutrophils eject chromatin with granular and nuclear proteins by a phagocytosis-independent mechanism before undergoing NETosis, a cell death process that is morphologically distinct from apoptosis and necrosis [11, 12, 13].

Although extracellular traps have beneficial effects, their ineffective clearance is known to cause host pathology. For example, impaired clearance of NETs is associated with aggravation of atherosclerosis [14], rheumatoid arthritis [15], and systemic lupus erythematosus (SLE) [16, 17, 18]. These responses are mediated by cytotoxic effect of NETs on host tissues. For example, extracellular histones and peptides in NETs were reported to induce epithelial and endothelial cell death [19]. Furthermore, the prolonged presence of NETs in the lungs may trigger autoimmune responses [20].

Recently, it was reported that macrophages also release extracellular traps [21, 22, 23, 24]. Macrophage extracellular traps (METs) were shown to capture and mediate clearance of bacteria, fungi and parasites [21, 24, 25]. MET formation by macrophages of various species was examined in the context of infection with different microbes. Human macrophages differentiated from peripheral blood monocytes induced extracellular trap formation against *Strongyloides stercoralis* [25]. *Histophilus somni* and *Mannheimia haemolytica* infection induced MET formation by bovine and murine macrophages [21, 22]. In these studies, MET formation was shown to be associated with activation of elastase or NADPH oxidase. In addition to direct contact with microbes, bacterial exotoxins also induce the formation of extracellular traps from macrophages [21]. For example, bovine monocyte-derived macrophages exposed to leukotoxin of *Mannheimia haemolytica* released METs that entrapped and killed bacteria.

A recent study reported that *M*. *tuberculosis*, a slow growing virulent mycobacteria, triggers not only cell death but also extracellular trap formation in neutrophils and macrophages [23, 26]. In this study, we show that *M*. *mass*, a rapid growing mycobacteria (RGM), also induces the release of extracellular traps from PMA-differentiated THP-1 macrophages. We examined the composition of *M*. *mass*-induced METs, and demonstrate that the mechanism of MET formation is dependent on calcium influx and phagocytosis. Our present results suggest that these METs not only entrap bacteria, but also perform complex roles in the interaction between host and rapidly growing, virulent mycobacteria.

## Materials and Methods

### Bacterial strains

A clinically isolated rough strain of *M*. *mass* (*M*. *mass* R), reported as *M*. *mass* Asan 50594 belonging to the Type II genotype, was used in this study [27, 28, 29]. *M*. *mass* type II genotype was reported to show loss of genes related to glycopeptidolipid (GPL) biosynthesis and rough type due to an irreversible genetic factor [29]. *M*. *mass* strain CIP 108297 (*M*. *mass* CIP) was obtained from CIP (Collection of Institut Pasteur, Paris, France). *M*. *mass* were cultured in Middlebrook 7H9 medium (BD Biosciences, Franklin Lakes, NJ, USA) supplemented with 10% OADC (BD Biosciences, Franklin Lakes, NJ, USA), 0.2% glycerol and 0.05% Tween 80 (Sigma-Aldrich, St. Louis, MO, USA). Cultured bacteria were collected by centrifugation. Then, collected mycobacteria were stored at -70°C until use. To prepare single cells of *M*. *mass*, cultured bacteria were homogenized with 23G syringe needle and sonicated in 10 min after soft spin centrifugation to exclude bacterial clumping. Nonsonicated *M*. *mass* aggregates were prepared by performing only soft spin centrifugation of the same bacterial cultures. The number of viable bacteria in stored bacterial vials was counted on Luria-Bertani (LB) agar (BD Biosciences, Franklin Lakes, NJ, USA).

### Cell culture and reagents

The human acute monocytic leukemia THP-1 cell line was maintained in RPMI media supplemented with 10% FBS (Gibco, Carlsbad, California, USA). Differentiation of THP-1 cells into macrophages was performed by incubation with 25 ng/ml phorbol 12-myristate 13-acetate (PMA) (Sigma-Aldrich, St. Louis, MO, USA) for 2 days at 37°C in a humidified atmosphere with 5% CO_2_. In these experiments, THP-1 cells were grown in 24-well tissue culture plates (Corning, Corning, NY, USA), and PMA-differentiated THP-1 macrophages were selected by keeping only the adherent cells.

### Quantification of extracellular trap

PMA-differentiated THP-1 cells were cultured on 12-mm glass cover slides in 24 well plates (2×10^5^ cells/well), and infected by *M*. *mass* R or CIP at multiplicity of infection (MOI) of 5, 10 or 20 (bacteria per cell) with or without 50 units/ml DNase I (Sigma-Aldrich, St. Louis, MO, USA), or stimulated with 1 mM hydrogen peroxide or 10 μg/ml LPS (Sigma-Aldrich, St. Louis, MO, USA) for 24 hr. To examine the responses of THP-1 macrophages infected with only intracellular *M*. *mass* R or CIP, infected cells were washed twice at 3hr after infection to eliminate extracellular bacteria, and cultured for 24hr. For fluorescence staining of *M*. *mass* R, the Vybrant CFDA-SE (CFSE) cell tracer kit (Thermo Fisher, Waltham, MA, USA) was used. To visualize extracellular traps, cells were stained with 1 μM TO-PRO-3 (Thermo Fisher, Waltham, MA, USA) for 30 min at 37°C, and examined using fluorescence microscope CTR6000 (LEICA, Wetzlar, GE). Macrophages releasing extracellular DNA structures were considered as producing METs. For quantification of MET production, the total number of macrophages and the number of macrophages releasing METs per field of view were counted in 4 or 5 individual images per sample. At least 400 cells per sample were examined and expressed as a percentage.

### Scanning Electron Microscopy (SEM)

*M*. *mass* CIP-infected cells on cover slips were washed, fixed with 2.5% glutaraldehyde overnight, and washed with 0.1 M phosphate buffer twice and post-fixed with 1% osmium tetroxide for 70 min. The samples were subsequently dehydrated with a graded ethanol series (30%, 50%, 70%, 80%, 90%, 100%), and then prepared as previously described [12]. The samples were examined using a scanning electron microscope (JEOL JSM-7401f, Japan).

### Immunofluorescence microscopy

To perform immunofluorescence staining, *M*. *mass* R-infected cells were fixed with 4% paraformaldehyde (PFA) for 15 min, and permeabilized with 0.1% Triton X-100 for 5 min and blocked in 1% BSA/PBS for 1 hr at room temperature. The samples were subsequently incubated with rabbit anti-histone H4, rabbit anti-myeloperoxidase (MPO) or rabbit anti-elastase antibody (Santa cruz, Dallas, TX, USA) at 1:50–1:100 dilution for 90 min at room temperature. They were then washed and incubated with Alexa 488-conjugated anti-rabbit IgG (Thermo Fisher, Waltham, MA, USA) at 1:200 dilution for 90 min. Lastly, the slides were stained with 1μM TO-PRO-3 for 30 min and examined using fluorescence.

### Cytokine treatment of macrophage

Differentiated THP-1 macrophages were pretreated with 10ng/ml IFN-γ (ProSpec, East Brunswick, NJ, USA) and TNF-α (R&D Systems, NE Minneapolis, MN, USA) or untreated for 24 hr. The cells were washed and infected with *M*. *mass* R or CIP (MOI 5) for 24 hr. Then, MET formation in each sample was examined.

### Cytokine analysis

For cytokine analysis, the supernatants of THP-1 macrophages infected with *M*. *mass* R or CIP (5 MOI) were harvested at 24hr post infection, and stored at -80°C until use. The levels of inflammatory cytokines (TNF-α, IL-1β and IL-6) were quantified using an enzyme-linked immunosorbent assay (ELISA) kit (Roche, Basel, Switzerland). The assays are performed according to the manufacturer’s instructions.

### Polymerase chain reaction (PCR)

After extracellular traps were induced by infection with *M*. *mass* R for 24 hr, released DNA in the supernatants of infected cells and uninfected control were purified using QIAamp MinElute Media Kit (Qiagen, Venlo, Netherlands). PCR analysis was performed on purified DNA using two nuclei-specific primers (Gapdh, Forward: 5’-AAT CCC ATC ACC ATC TTC CA-3’, reverse: 5’-TGG ACT CCA CGA CGT ACT CA-3’. β-actin, Forward: 5’-GTT GCT ATC CAG GCT GTG-3’, reverse: 5’- TGA TCT TGA TCT TCA TTG TG-3’) and two mitochondria-specific primers (Atp6, Forward: 5’-ATA CAC AAC ACT AAA GGA ACC T-3’, reverse: 5’-GAG GCT TAC TAG AAG TGT GAA AAC G-3’. Nds1, Forward: 5’-GCA TTC CTA ATG CTT ACC GAAC-3’, reverse: 5’-AAG GGT GGA GAG GTT AAA GGA G-3’). PCR reaction was carried out using a MyCycler^TM^ (Biorad, Hercules, CA, USA). Initial denaturation at 95°C for 10 min was followed by 28 cycles of denaturation at 94°C for 30 sec, annealing at 58°C for 40 sec, and extension at 72°C for 90 sec, with a final incubation for extension at 72°C for 7 min. The PCR products were analyzed by 1.5% agarose gel electrophoresis.

### Reactive Oxygen Species (ROS) Detection

Differentiated THP-1 macrophages were pretreated with 20 μM DPI or left untreated for 30 min before stimulation with 1 mM hydrogen peroxide or *M*. *mass* R (MOI 5) for 24 hr. The cells were washed and stained with intracellular indicator, 5 μM 2’, 7 dichlorofluorescein diacetate (H2DCF-DA) (Thermo Fisher, Waltham, MA, USA) for 30 min. After washing, labeled cells were treated with 5 mM EDTA for 15 min, and collected. The samples were subsequently washed and analyzed using BD FACSCalibur and data analysis was done using FlowJo software (BD Biosciences, Franklin Lakes, NJ, USA).

### Inhibition of macrophage elastase by N-Methoxysuccinyl-Ala-Ala-Pro-Val p-nitroanilide (AAPV)

Differentiated THP-1 macrophages were untreated or pretreated with AAPV (0.1 or 1 μM) (Sigma-Aldrich, St. Louis, MO, USA) for 30 min before infection with *M*. *mass* R (MOI 5) for 24 hrs. The MET formation in each sample was quantified as previously described.

### Measurement of intracellular calcium influx

For monitoring intracellular calcium influx, THP-1 cells were plated at 5 x 10^4^ cell/well in 96-well black microplates (Corning, Corning, NY, USA) and differentiated by PMA. Differentiated THP-1 cells were washed and loaded with the Ca^2+^-sensitive indicator, 1 μM Fluo-4 AM (Thermo Fisher, Waltham, MA, USA) for 20 min. Then, Fluo-4-labelled cells were untreated or pre-treated with 20 μM 1,2-bis(o-aminophenoxy)ethane-N,N,N',N'-tetraacetic acid (BAPTA) (Thermo Fisher, Waltham, MA, USA) or 1 μM ethylene glycol tetraacetic acid (EGTA) for 30 min, and subsequently infected with *M*. *mass* R (MOI 5) or stimulated with 1 μM ionomycin (Sigma-Aldrich, St. Louis, MO, USA) as positive control. Fluo-4-labelled cells were washed at 3 hr post infection and measured at 494nm/516nm using a microplate reader (Tecan infinite 200, San Jose, CA, USA). Fluorescence intensity was measured at 20 sec intervals over 10 min.

### Assessment of bacterial survival and phagocytosis

To assess bacterial survival in the presence or absence of METs, cells were pre-treated with 50 unit/ml DNase I (Sigma-Aldrich, St. Louis, MO, USA) for 30 min, and then infected by *M*. *mass* R (MOI 10) for 24 hr to induce MET release. The supernatants were collected to recover extracellular bacteria, and attached cells with METs were gently washed and lysed with PBS containing 0.1% Triton X-100 (Sigma-Aldrich, St. Louis, MO, USA) to recover cell-associated bacteria. To determine the extent of phagocytosis of bacteria, cells were or were not pre-treated with 5 μM Cytochalasin D (Sigma-Aldrich, St. Louis, MO, USA) for 30 min, and then infected by *M*. *mass* R or CIP (MOI 1, 3 or 10) for 24 hr to induce MET release. Infected cells were washed several times and lysed to recover only intracellular bacteria. The supernatants or released bacterial suspension was diluted in PBS, and plated on LB agar for CFU determination. The summation of CFUs recovered from cells and supernatants reflect the total quantities of surviving bacteria in each well. To examine *M*. *mass* within macrophages, acid-fast bacilli (AFB) stain kits (BD Biosciences, Franklin Lakes, NJ, USA) were used.

### Lactate dehydrogenase (LDH) release assay

To assess cell lysis in infected cells, PMA-differentiated THP-1 cells were cultured in 24 well plates (2×10^5^ cells/well), and infected by *M*. *mass* R (MOI 1, 3, or 10) for 24 hr. The supernatants were collected and Lactate dehydrogenase (LDH) release detection was performed using cytotoxicity detection kit (Roche, Mannheim, Germany) according to the manufacturer's instructions.

### Annexin V and Propidium iodide staining for flow cytometry

To determine necrotic cell death, differentiated THP-1 macrophages were infected with *M*. *mass* R (MOI 5) or left uninfected for 1 day. Collected cells were washed and then stained with Annexin V FITC (BD Biosciences, Franklin Lakes, NJ, USA) and Propidium iodide (BD Biosciences, Franklin Lakes, NJ, USA) for 15 min. Stained cells were analyzed using BD LSR Fortessa (BD Biosciences, Franklin Lakes, NJ, USA), and data analysis was done using FlowJo software (BD Biosciences, Franklin Lakes, NJ, USA).

### Statistical analyses

ANOVA and Student’s *t*-test were performed to determine statistically significant differences between groups using GraphPad Prism (GraphPad Software, La Jolla, CA, USA). A *P* value of < 0.05 was deemed to be statistically significant and is indicated in the figures by an asterisk. *P* values of <0.01 and <0.001 are indicated by two and three asterisks, respectively.

## Results

### Microscopic examination of MET formation induced by *M*. *mass* infection

**Fig 1:**
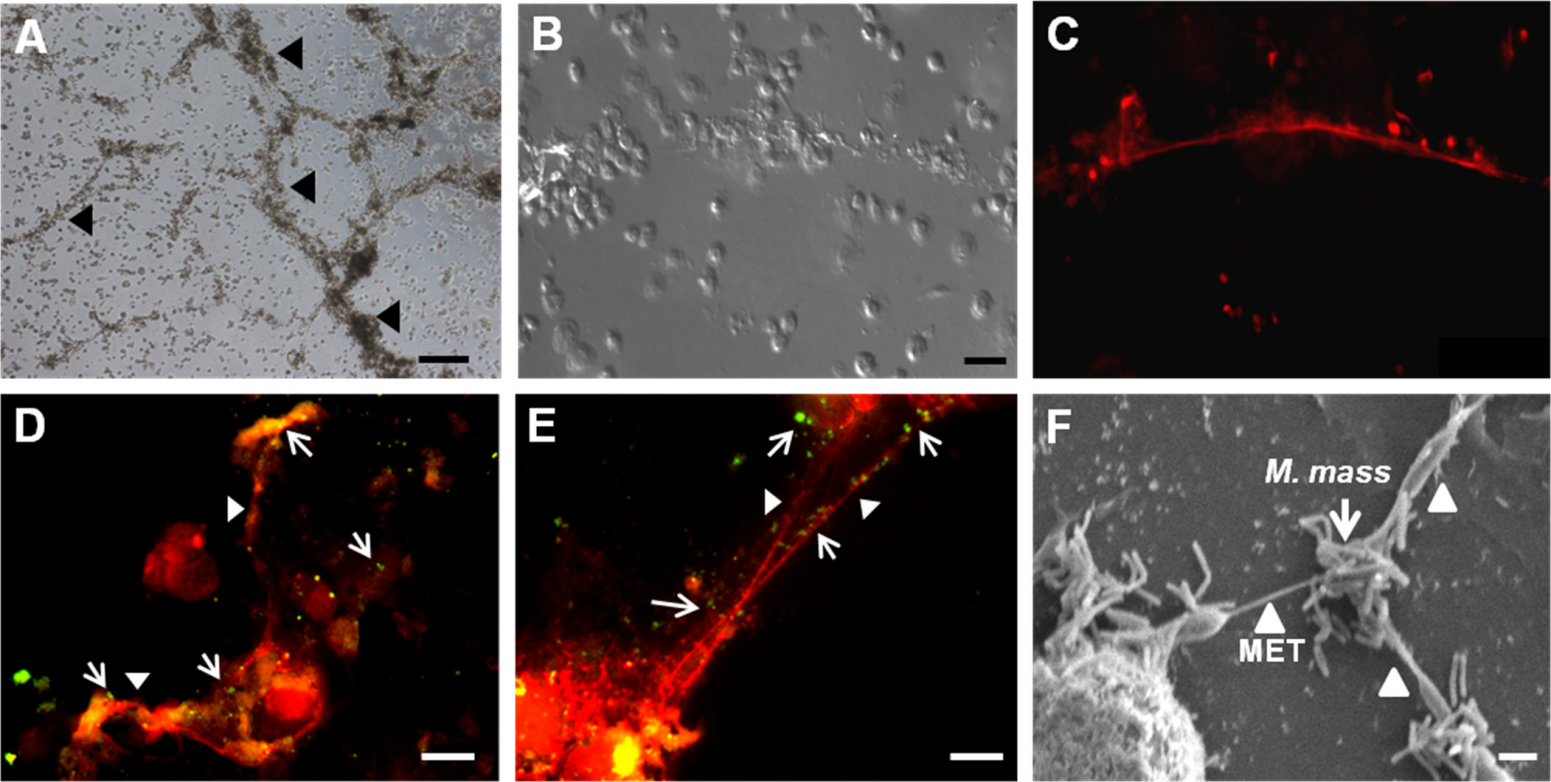
Microscopic examination of macrophage extracellular trap (MET) formation induced by *M*. *mass* infection. Differentiated THP-1 macrophages were infected with *M*. *mass* R or CIP at a MOI of 5 for 24 hr, and stained by DNA-binding dye, TO-PRO-3 (Red) to visualize extracellular trap-like structure. (A) Microscopic examination of extracellular trap-like structures in *M*. *mass* R-infected THP-1 macrophages. Bar, 200μm. (B, C) Microscopic examination (B) and TO-PRO staining (C) of extracellular trap-like structures induced by *M*. *mass* R infection. Bar, 50μm. (D, E) Fluorescence micrographs of CFSE-stained *M*. *mass* R (Green) entrapped by METs. Bar, 20μm. (F) Scanning electron microscopy (SEM) images of METs induced by *M*. *mass* CIP. Bar, 1μm. Arrow: Mycobacteria. Arrow heads: Extracellular trap-like structures.

In a previous study, *M*. *abscessus* infection induced the formation of DNA extracellular trap structures by human peripheral blood mononuclear cells (PBMC) [30]. Similarly, when PMA-differentiated THP-1 macrophages were infected by *M*. *mass* cells released extracellular fiber structures that merged together (Fig 1A). These structures were positively stained by a DNA-staining dye, TO-PRO, and were morphologically similar to DNA extracellular traps (Fig 1B and 1C). We observed similar structures in PBMC-derived human macrophages infected with *M*. *mass* (data not shown). To confirm whether these structures can function like METs and entrap bacteria, we infected macrophages with CFSE-stained *M*. *mass*. Fluorescence microscopic examination revealed CFSE-stained *M*. *mass* co-localization with extracellular DNA fibers 1 day after infection, suggesting that extracellular *M*. *mass* was entrapped and bound to the MET structures (Fig 1D and 1E). In addition, we used scanning electron microscopy (SEM) to examine the ultrastructure of the METs induced by *M*. *mass* (Fig 1F). In SEM examination, most of extracellular *M*. *mass* were shown to be attached to macrophages. Some mycobacteria were linked to METs released from adjacent macrophages. MET structures were more common near clustered *M*. *mass* rather than single or a few bacilli. Thus, we confirmed that *M*. *mass* infection induces the formation of DNA extracellular traps from differentiated THP-1 macrophages and that *M*. *mass*-induced METs are morphologically similar to the extracellular structures released by activated neutrophils and other immune cells.

### *M*. *mass* with rough colony morphology strongly induces MET formation

**Fig 2:**
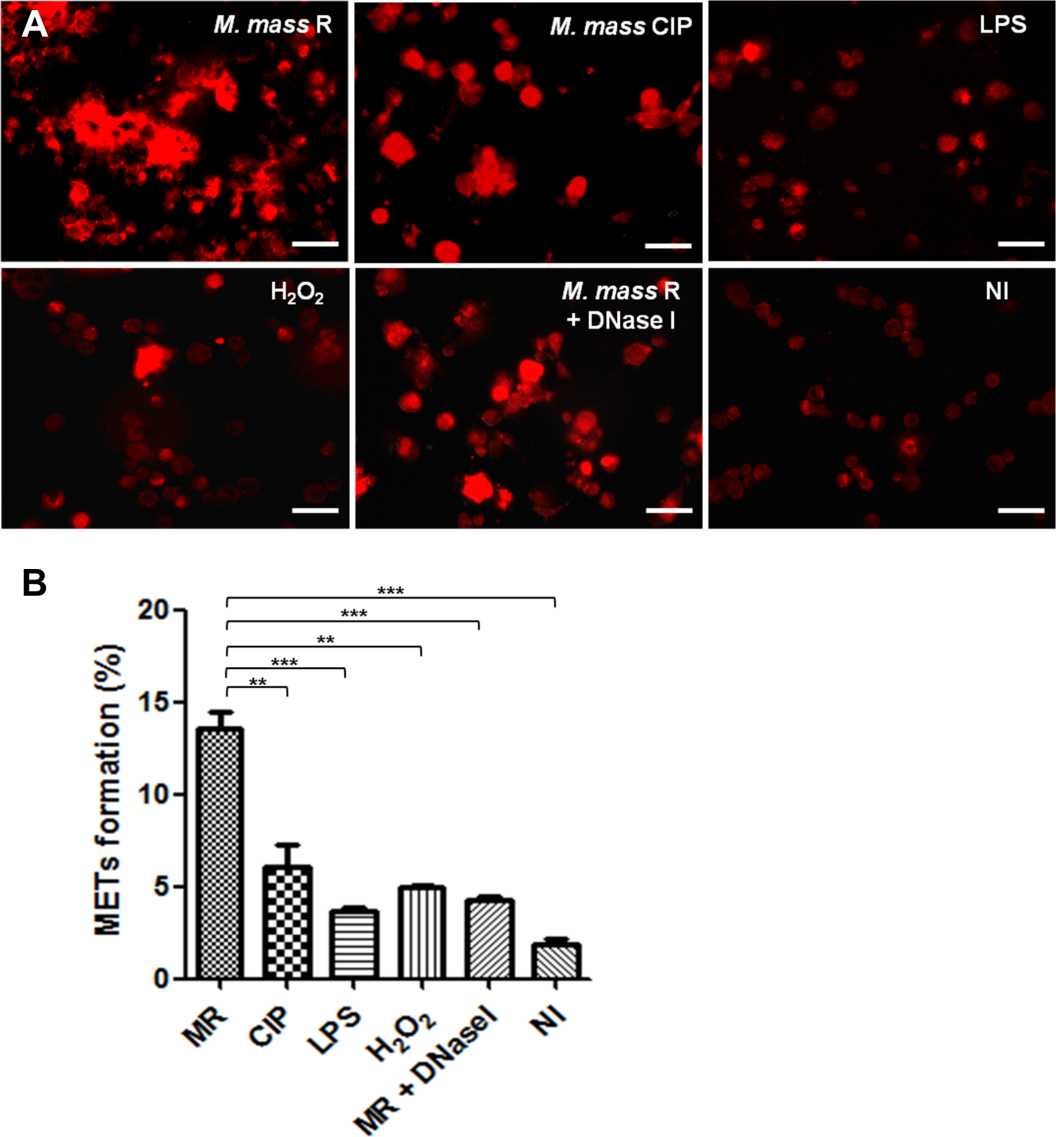
Rough strain of *M*. *mass* strongly induces MET formation in differentiated THP-1 macrophages. (A) Differentiated THP-1 macrophages were stimulated with *M*. *mass* R (MOI 20) with or without DNase I (50 units/ml), *M*. *mass* CIP (MOI 20), LPS (10 μg/ml) or hydrogen peroxide (1 mM) for 24 hr, and then stained by TO-PRO-3 to examine METs. Bar, 50μm. (B) MET formation (%) of each sample was quantified by calculating percentages of MET-positive cells to total cells count. MR: *M*. *mass* R. Data are representative of three independent experiments with similar results. *, p<0.05; **, p<0.01 compared to *M*. *mass* R-infected group by one-way ANOVA with Bonferroni’s post-test.

Various stimuli have been shown to induce neutrophil extracellular traps including PMA, LPS, hydrogen peroxide, etc [6, 31]. We examined the effects of such stimuli on inducing MET formation by PMA-differentiated THP-1 macrophages (Fig 2A). LPS (10 μg/ml) and hydrogen peroxide (1 mM) treatments for 1 day did not induce extracellular traps from macrophages, and high concentrations of stimuli (LPS ≥ 100 μg/ml; H_2_O_2_ ≥ 10mM) caused extensive cell death. In addition, we infected THP-1 macrophages with two different strains of *M*. *mass*, those that form rough and smooth colonies, to determine whether colony type affects MET formation or not. We infected the cells with each *M*. *mass* at 20 MOI, to induce extreme responses by each colony type for 1 day and compare them with the effects of other stimuli. After infecting macrophages with each strain of *M*. *mass*, the sample infected with the rough strain, *M*. *mass* R, showed MET formation in 14% of the cells (Fig 2B). On the other hand, less than 6% of the macrophages infected by the smooth strain, *M*. *mass* CIP, formed METs. This result is similar to that of previous study using human monocytes infected with *M*. *abscessus* [30]. We examined whether only intracellular infection without extracellular bacteria induces MET formation. The sample with only intracellular infection showed much lower levels of MET formation than the sample infected with both intracellular and extracellular bacteria (S1 Fig). DNase treatment during infection readily dissolved the extracellular traps, but *M*. *mass*-induced cell death during MET formation was still detected.

*M*. *mass* R significantly induced MET formation. However, when THP-1 macrophages were infected with *M*. *mass* R at MOI of 20, severe cell death was detected 24 hr after infection. So we performed the following experiments using *M*. *mass* R or CIP at MOI of 5. To examine the effect of bacterial aggregation on MET formation, we infected THP-1 macrophages with sonicated single cells or nonsonicated aggregates of *M*. *mass*. Colony-forming unit (CFU) of nonsonicated *M*. *mass* were measured half as many as that of sonicated *M*. *mass*. When macrophages were infected by 5 MOI of sonicated or 2.5 MOI of nonsonicated *M*. *mass* R, nonsonicated aggregates of *M*. *mass* R induced more MET formation and cell death than sonicated single bacteria did. However, nonsonicated *M*. *mass* CIP did not enhance the levels of MET formation or cell death, comparing with sonicated *M*. *mass* CIP (S2 Fig). Previous studies reported that rough strains of *M*. *mass* induce higher levels of proinflammatory cytokines than smooth strains. Our study also showed that *M*. *mass* R induce higher levels of TNF-α, IL-1β and IL-6 than *M*. *mass* CIP (S3A Fig). However, pretreatment of macrophages with IFN-γ or TNF-α did not enhance MET formation by infection with *M*. *mass* R or CIP (S3B Fig). These results suggest that DNA extracellular trap production by macrophages is not due to simple inflammatory or oxidative stimuli, but is dependent on the colony type of *M*. *mass*, indicating that interactions with distinct antigens or surface structures of *M*. *mass* may cause the release of extracellular traps from macrophages.

### *M*. *mass* R-induced METs contain mitochondrial DNA, nuclear DNA, and microbicidal proteins

**Fig 3:**
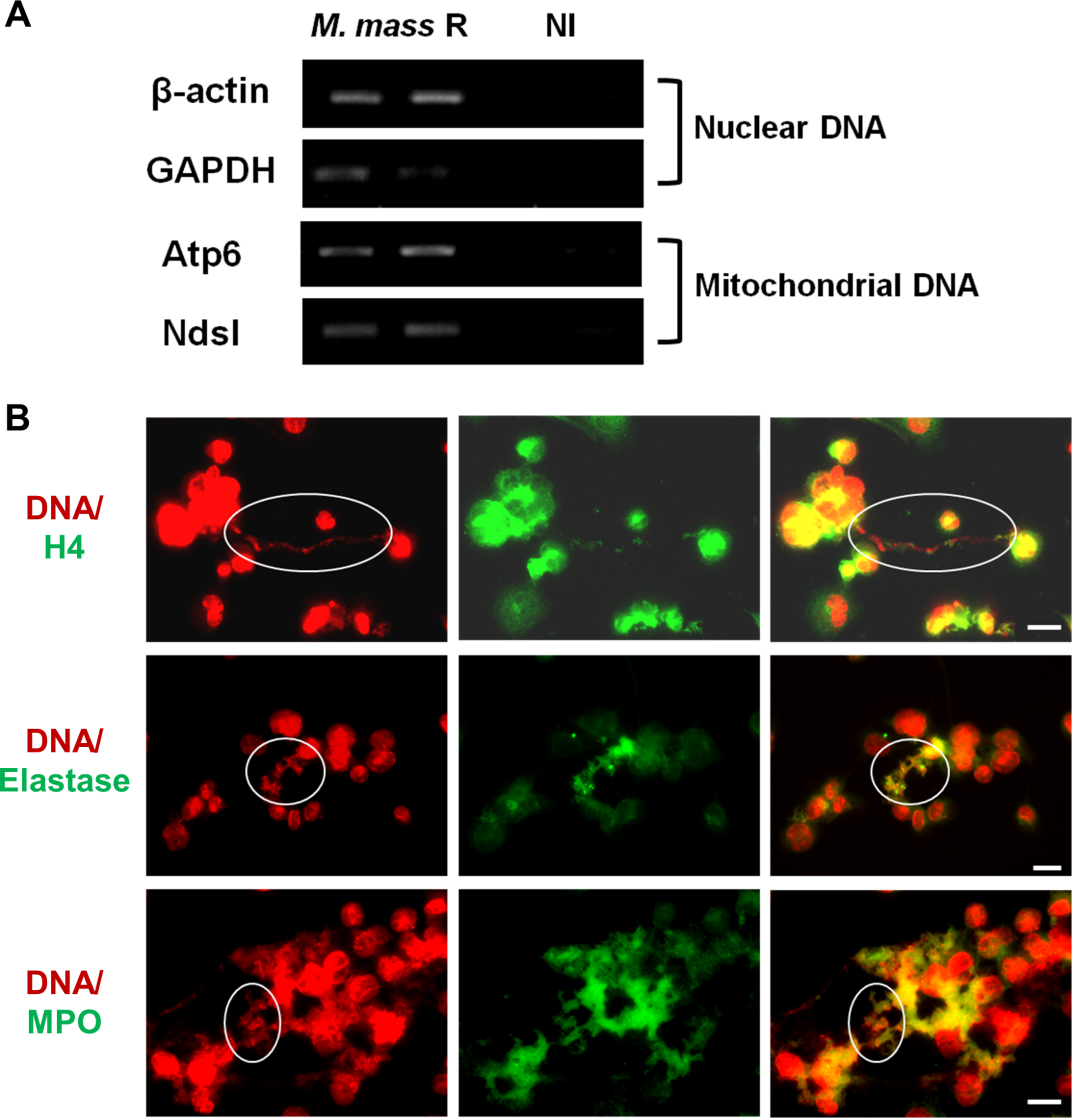
*M*. *mass* R-induced METs induce release of mitochondrial as well as nuclear DNA, and contain Histone, MPO and Elastase. (A) PCR analysis was performed for nuclear (*β-actin*, *Gapdh*) and mitochondrial genes (*Atp6*, *Nds1*) using DNA isolated from the supernatants of *M*. *mass* R-infected THP-1 macrophages and uninfected control samples. Both nuclear and mitochondrial genes were detected in the infected samples. Data are representative of three independent experiments. (B) *M*. *mass* R-infected THP-1 macrophages were fixed and processed for histone 4, elastase and MPO staining. METs were stained by TO-PRO-3. Circles indicate co-localization of METs with each component. Data are representative of three independent experiments. H4: histone 4. Bar, 20μm.

Extracellular traps formed by different immune cells are known to consist of nuclear or mitochondrial DNA embedded with histones and various enzymatic peptides [6, 16, 32]. To investigate the origin of the DNA released by macrophages infected with *M*. *mass*, we infected THP-1 cell with *M*. *mass* R and performed PCR analysis to detect nuclear signals (β-Actin and Gapdh) and mitochondrial signals (Atp6 and Nds1). PCR analysis of the supernatants from *M*. *mass* R-infected cells, containing visible MET structures, showed that the extracellular DNA contained sequences of both nuclear and mitochondrial genes (Fig 3A). It is possible that the extracellular DNA in supernatants is due to the release of cell contents from cell death associated with *M*. *mass* R-induced MET production. In addition, we performed immunofluorescence staining to identify the DNA-embedded components in *M*. *mass* R-induced METs (Fig 3B). We used specific antibodies to test for potential candidates such as histones and antimicrobial enzymes (MPO and elastase), and found that histones colocalized with the extracellular DNA of METs while antimicrobial enzymes colocalized with METs. The latter suggests that *M*. *mass* R-induced METs also have enzyme-mediated bactericidal functions. However, we also observed that portions of METs did not contain histones or enzymes. Collectively, we show that *M*. *mass* R-induced METs have a backbone of nuclear and mitochondrial DNA that is embedded with histones and enzymatic proteins, suggesting they have similar functions in host defense against foreign pathogens to extracellular traps of other immune cells.

### *M*. *mass* R-induced MET formation does not depend on NADPH oxidase, but rather calcium influx

**Fig 4:**
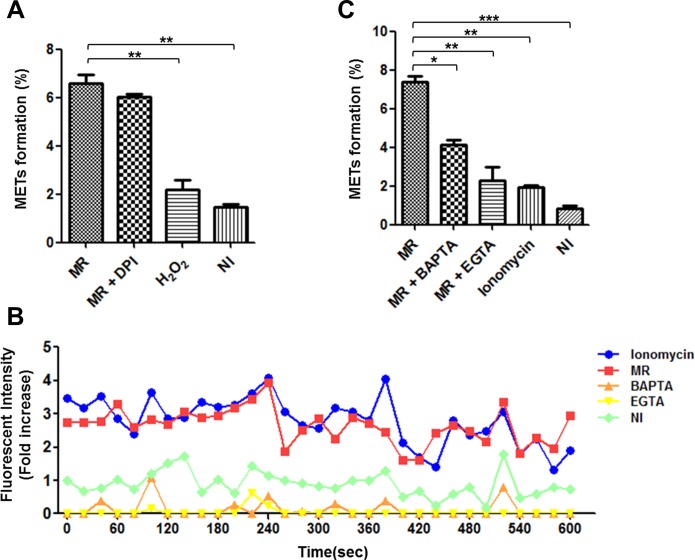
*M*. *mass* R-induced MET formation is not dependent on NADPH oxidase activity, but does depend on calcium influx. After staining THP-1 macrophages with TO-PRO-3, the MET formation (%) in each sample was determined by quantification of MET-positive cells to total cell count. (A) Differentiated THP-1 macrophages were stimulated with hydrogen peroxide (1 mM), *M*. *mass* R (MOI 5) with or without DPI (20 μM) for 24 hr. (B) Fluo-4-labeled THP-1 macrophages were untreated or pretreated with BAPTA (20 μM) or EGTA (1 mM) for 30 min and then infected with *M*. *mass* R (MOI 5). Ionomycin (1 μM) was used as positive control. Intracellular calcium transients of labeled cells were recorded in a microplate reader at 6 hr after infection. (C) Determination of MET formation (%] in each sample. MR: *M*. *mass* R. Data are representative of three independent experiments. *, p<0.05; **, p<0.01; ***, p<0.001 compared to *M*. *mass* R-infected group by one-way ANOVA with Bonferroni’s post-test.

Extracellular trap formation is known to be closely associated with the activation of NADPH oxidase [6, 33, 34]. To investigate whether NADPH oxidase is required for *M*. *mass* R-induced MET formation, we treated infected macrophages with diphenylene iodonium (DPI), an inhibitor of NADPH oxidase enzymes (Fig 4A). Although treatment of infected macrophages with DPI reduced ROS production, *M*. *mass* R infection did not augment ROS production compared to uninfected cells (S4 Fig). Microscopic examination showed that DPI treatment of *M*. *mass* R-infected cells did not significantly reduce MET formation, suggesting that inhibition of ROS generation by NADPH oxidase does not affect extracellular trap release from macrophages by *M*. *mass* R infection. Although hydrogen peroxidase treatment enhanced ROS generation in macrophages, it did not induce MET formation. In a previous study using *M*. *tuberculosis*, MET formation was reported to be associated with elastase activity [26]. We tested using various concentrations of N-Methoxysuccinyl-Ala-Ala-Pro-Val p-nitroanilide (AAPV), elastase inhibitor, but it did not affect *M*. *mass* R-induced MET formation (S5 Fig).

Recent studies have reported that calcium influx also triggers the formation of extracellular traps via an NADPH oxidase-independent mechanism [35, 36]. To investigate the role of calcium influx in *M*. *mass* R-induced MET formation, we measured calcium influx in infected and uninfected THP-1 macrophages by staining cells with Fluo-4 AM (Fig 4B). Fluorescence intensity measurement showed that *M*. *mass* R infection triggers a slow increase in the level of intracellular calcium that apparently exceeds uninfected cells at 6 hr after infection. The chelation of intracellular or extracellular calcium using BAPTA or EGTA, respectively, decreased calcium influx in *M*. *mass* R-infected macrophages. To evaluate the effects of calcium influx on METs formation, we monitored the formation of extracellular traps by *M*. *mass* R-infected macrophages after pretreatment with BAPTA or EGTA (Fig 4C). Although both treatment with BAPTA and EGTA reduced intracellular calcium levels of infected cells at early stages, however, BAPTA and EGTA did not completely abolish MET formation. Conversely, although treatment with ionomycin increased calcium influx, it could not trigger significant release of METs. Collectively, these results suggest that production of extracellular traps is partially affected by calcium-mediated inflammatory signals induced by *M*. *mass* R infection, and not calcium influx, in an NADPH oxidase-independent manner.

### MET formation is dependent on phagocytosis of *M*. *mass* R

**Fig 5:**
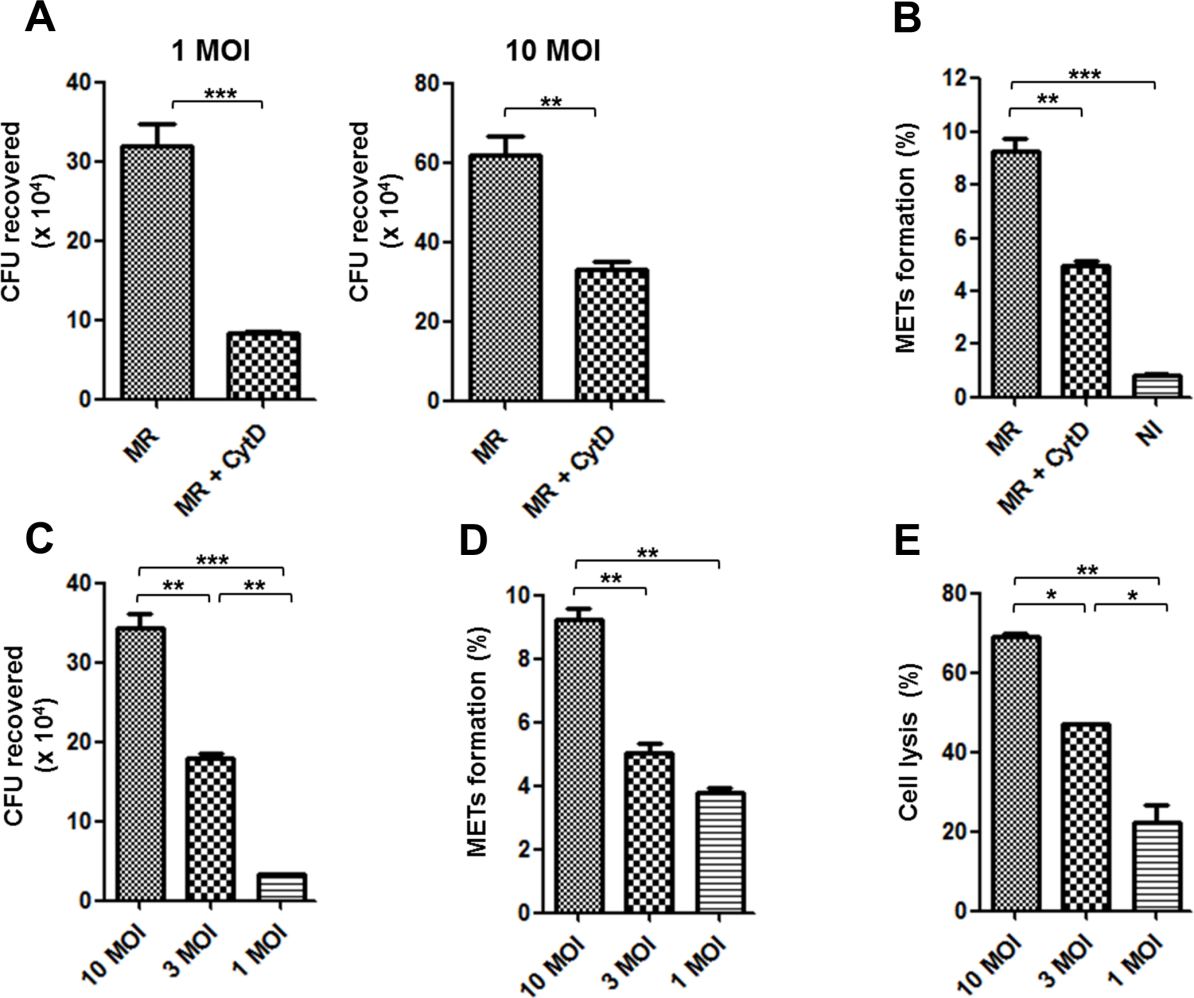
MET formation is dependent on phagocytosis and cell lysis. (A) CFUs of *M*. *mass* R recovered from infected macrophages with or without cytochalasin D (5 μM) for 24 hr. (B) MET formation (%) in the macrophages infected with *M*. *mass* R (MOI 10) after pretreatment with or without cytochalasin D. (C) CFUs recovered from macrophages infected with *M*. *mass* R at various MOI at 4 hr post infection. (D) Determination of MET formation (%) in each sample at 24 hr post infection. (E) Supernatants were collected from macrophages infected with *M*. *mass* R at various MOIs at 24 hr post infection. The level of cell lysis of each group was determined by LDH release assay. MR: *M*. *mass* R. Data are representative of three independent experiments. ns, non-significant; *, p<0.05; **, p<0.01; ***, p<0.001 by Student’s *t*-test (A) or one-way ANOVA with Bonferroni’s post-test (B-E).

Considering the previous results, we supposed that an intracellular direct interaction between macrophages and *M*. *mass* R may contribute to MET formation. To analyze the dependency of MET formation on phagocytosis of *M*. *mass* R, we pretreated THP-1 macrophages with a phagocytosis blocker, cytochalasin D (CytD), prior to *M*. *mass* R infection and analyzed changes in MET formation. Treated cells showed decreased phagocytosis, represented by decreased intracellular CFUs, compared to untreated cells (Fig 5A). This was correlated with reduced MET formation in CytD-treated cells, suggesting that induction of MET is dependent on phagocytosis of *M*. *mass* R (Fig 5B). To corroborate this, we investigated MET formation after phagocytosis of different numbers of bacteria (Fig 5C). We infected the cells with *M*. *mass* R at 10, 3 and 1 MOI, to examine gradually induced MET formation and cell lysis for 1 day. Indeed, the extent of MET formation depended on the initial amount of bacteria that was phagocytosed (Fig 5D). In addition, LDH release data showed that cell lysis occurred after infection with *M*. *mass* R in a manner that is also correlated with bacterial burden (Fig 5E). Necrotic cell death of macrophages was confirmed by Annexin V and PI staining (S6 Fig). To examine whether *M*. *mass* CIP induce similar responses after cell lysis, we infected macrophages with *M*. *mass* CIP at 10, 3 and 1 MOI. *M*. *mass* CIP did not induce a significant increase in MET formation and cell lysis for 1 day (data not shown). At 2 day after infection, while *M*. *mass* R-infected cells are almost dead (data not shown), *M*. *mass* CIP-infected macrophages underwent gradual cell death responses depending on MOIs (S7A Fig). However, the samples infected with *M*. *mass* CIP for 2 days showed lower levels of MET production, comparing with the samples infected with the same amount of *M*. *mass* R for 1 day (S7B Fig). Taken together, these results suggest that MET formation is associated with macrophage phagocytosis of *M*. *mass* R, rather than an extracellular interaction with bacteria. Moreover, *M*. *mass* R induces more active cell necrosis and MET formation than *M*. *mass* CIP, and the subsequent responses associated with cell necrosis seem to contribute to the release of DNA.

### METs have no bactericidal effects on *M*. *mass* R

**Fig 6:**
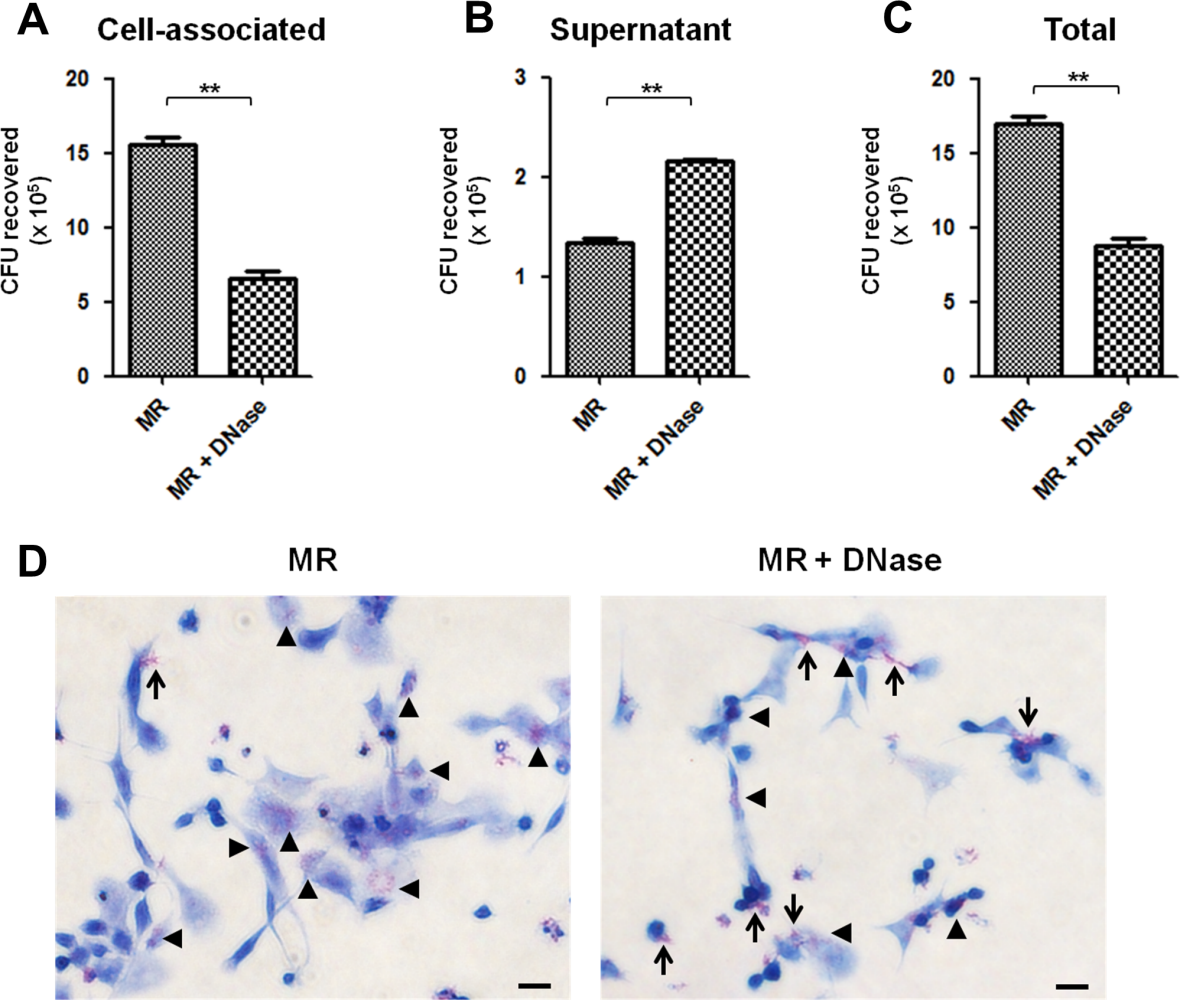
Released METs have no bactericidal effects on *M*. *mass* R. To determine the bactericidal effect of METs on *M*. *mass* R, macrophages with METs-degrading DNase I 30 min before infection. (A) Cell-associated (intracellular or METs-bound), (B) Extracellular, (C) Total number of bacteria recovered from the samples infected by *M*. *mass* R (MOI 5) for 24 hr, with or without DNase (50 units/ml). (D) AFB staining of THP-1 macrophages infected by *M*. *mass* R with or without DNase for 24 hr. Bar, 20μm. Data are representative of three independent experiments with similar results. **, p<0.01 by Student’s *t*-test.

Extracellular traps released from neutrophils or other immune cells are known to be microbicidal by entrapping microbes [6, 8, 21]. To determine whether METs induced by *M*. *mass* R infection have anti-bacterial activity, we examined bacterial survival after treatment of infected macrophages with DNase to degrade METs. Interestingly, disintegration of METs by DNase caused a decrease in cell-associated *M*. *mass* R compared to untreated macrophages (Fig 6A). On the other hand, there was an increase in extracellular bacteria recovered from culture supernatant in DNase-treated samples (Fig 6B). These results suggest that METs have a role in entrapping microbes and thereby reduce extracellular CFUs in the supernatant, but may not have direct bactericidal activity on *M*. *mass* R. The collective amounts of *M*. *mass* R recovered from DNase-treated groups were significantly higher than those of untreated groups (Fig 6C). A previous study investigating METs induced by *M*. *tuberculosis* reported similar results that bacterial aggregation and growth increased as MET formation was enhanced [23]. To test whether *M*. *mass* R aggregation was induced by METs, we performed AFB stain on infected cells (Fig 6D). Indeed, we observed intracellular growth and aggregation of *M*. *mass* R were decreased in DNase-treated samples. Instead, extracellular bacteria attached to cell surface were detected more often in the same samples. These data indicate that METs have no bactericidal activity on *M*. *mass* R, but rather may increase MET-mediated interaction of bacteria with cell and facilitate stable aggregation of entrapped *M*. *mass* R.

## Discussion

Macrophages are distributed through-out many body tissues and contribute to tissue homeostasis and immune defense against foreign agents. Upon infection by pathogens, macrophages generally perform the first innate immune responses, engulfing and killing pathogens, and evoking subsequent responses by other immune cells. Recent studies report that macrophages can generate extracellular trap structures consisting of DNA as a backbone with microbicidal proteins, and entrap extracellular bacteria, fungi, and parasites [22, 24, 25]. Similar to extracellular traps of other immune cells, METs are known to be induced by various stimuli, such as microbes, microbial products or cytokines [21, 37]. However, the characteristics and host defense mechanisms of METs remain to be elucidated.

Investigations on the interactions of macrophages with mycobacteria previously focused on intracellular responses after phagocytosis [38, 39]. However, *M*. *tuberculosis* was recently shown to induce extracellular trap formation by human PBMC-derived macrophages, providing evidence of a novel role of macrophages in extracellular immune defense against mycobacterial infection [23]. In this study, we showed that *M*. *mass*, a rapid growing virulent mycobacterium, also induces the formation of extracellular traps from PMA-differentiated THP-1 macrophages. Notably, *M*. *mass* R, a rough isolate of *M*. *mass*, significantly induced more MET release than a smooth isolate of *M*. *mass* (Fig 2). The main difference between the rough and smooth strains is the loss of glycopeptidolipid (GPL) in cell surface, caused by loss or mutation of the genes related to GPL biosynthesis [29]. Rough strains are known to be more virulent and associated with acute respiratory disease [40, 41, 42]. Because loss of GPL unmasks bioactive cell wall lipids, rough strains induce higher inflammatory reponses than smooth strains [43, 44]. This mechanism is considered to be associated with MET formation. Similarly, in a previous study, the cord-forming rough isolate of *M*. *abscessus* was found to mainly induce extracellular trap formation by human monocytes [30]. In addition, nonsonicated aggregates of *M*. *mass* R was shown to induce more MET formation comparing sonicated single bacteria. This response was also examined in human macrophages infected with *M*. *tuberculosis* [23]. Collectively, these results indicate colony type and bacterial structures modulate interaction with macrophages and extracellular trap formation.

Previous study using *M*. *tuberculosis* showed IFN-γ pretreatment of human macrophages readily induced rapid necrotic death and MET formation [23]. These responses were strictly dependent on ESX-1. However, our results showed that pretreatment of THP-1 macrophages with IFN-γ or TNF-α do not enhance MET formation. Because *M*. *mass* do not have ESX-1 secretion system, *M*. *mass*-induced MET formation may be affected by not pretreatment with proinflammatory cytokines but other mechanisms. In addition, chemical agents, such as PMA, LPS or hydrogen peroxide, did not stimulate MET release by human THP-1 macrophages (Fig 2), indicating that extracellular trap release from macrophages is closely associated with signal transductions mediated by specific microbes or antigens [21, 23, 24].

The formation of extracellular traps by neutrophils or other immune cells is known to mainly depend on NADPH oxidase [6, 33, 34]. However, our study revealed that the activity of NADPH oxidase did not affect *M*. *mass*-induced MET formation (Fig 4A). A previous study reported similar results that *E*. *coli* and *Candida albicans* induce MET production by murine macrophages in an NADPH oxidase-independent manner [24]. On the other hand, another study using bovine macrophages showed that *Mannheimia haemolytica*-induced MET formation is dependent on the activation of NADPH oxidase [21]. Collectively, these data indicate that the requirements for NADPH oxidase and therefore mechanisms for NET and MET extracellular trap formation differ depending on the stimulus [45].

Recent studies suggest that calcium influx is a major regulator of NOX-independent NETosis and that calcium activated NETosis is induced faster than NOX-dependent NETosis [35, 36]. *M*. *mass* infection indeed increased the levels of intracellular calcium and MET formation was partially dependent on calcium influx. These results seem to be consistent with *M*. *tuberculosis*, which caused calcium influx and extracellular trap formation in neutrophil by secretory antigen, ESAT-6-mediated cytolysis [46]. In addition, calcium-activated NET formation is known to be associated with mitochondrial ROS and activation of the MAPK pathway [35]. However, such mechanisms have yet to be confirmed in MET formation induced by bacterial infection. Further studies are needed to elucidate the molecular mechanism of calcium-mediated MET formation.

Neutrophils generally release extracellular traps toward extracellular microbes, such as *Shigella flexneri* and *Staphylococus aureus*, in a phagocytosis-independent manner [6]. However, a recent study reported *M*. *tuberculosis*-induced NET formation depends on phagocytosis [47]. In the study, *M*. *tuberculosis*-infected neutrophils after pretreatment with CytD closely resembled the uninfected control. Similarly, *M*. *mass*-induced MET formation was reduced by inhibition of phagocytosis with CytD, suggesting that phagocytosis of *M*. *mass* by macrophages is required for the production of extracellular traps. A previous study reported similar results that CytD-treated bovine macrophages showed a decrease in METs formation in response to *M*. *haemolytica* infection [21]. It was also shown that infection of human macrophages with high burden of *M*. *tuberculosis* is linked to more extracellular trap formation [23]. These results suggest that extracellular trap production by macrophages mainly depends on inflammatory responses following phagocytosis of *M*. *mass*.

NET-associated cell death, designated as NETosis, is a process that is morphologically and functionally distinct from apoptosis or necrosis [11, 13]. A previous study reported that neutrophils release nuclear DNA while maintaining membrane integrity and phagocytic responses without cell lysis [13]. However, unlike NETosis, necrotic cell death was observed during MET formation after phagocytosis of *M*. *mass* R. Similarly, *M*. *tuberculosis* also induced MET formation via their secretory system ESX-1-dependent macrophage necrosis [23]. However, although *M*. *mass* CIP induced macrophage cell death, infected cells showed low levels of MET formation. Collectively, these results reveal that *M*. *mass*-induced MET formation is accompanied by necrotic cell death, which is different from NETosis and may be affected by specific antigens related to bacterial strains [21, 23, 24].

Embedded histones and antimicrobial enzymes in NETs are known to have microbicidal effects on entrapped microbes [6, 48, 49]. However, a recent study reported that some microbes, such as *Streptococcus pneumoniae* or *S*. *pyogenes*, have mechanisms to evade entrapment and destruction by extracellular traps [50, 51]. In our study, METs released from macrophages did not show bactericidal activity on *M*. *mass*, but rather enhanced bacterial growth. Previous study investigating METs induced by *C*. *albicans* also report similar result [24]. In another study, survival and growth of *M*. *tuberculosis* were not inhibited by METs, and bacterial aggregation and burden increased as extracellular trap formation from human macrophages was enhanced by stimulation with IFN-γ [23]. We also found that *M*. *mass* growth was enhanced in macrophages with METs. A recent study reported macrophages are able to process extracellular trap via the phagocytosis route [52]. In this regard, we consider entrapping by METs may enhance phagocytosis of *M*. *mass* by macrophages and bacterial aggregation, which may facilitate mycobacterial survival and growth. However, the mechanism for evading the bactericidal action of METs and for enhancing mycobacteria survival is not yet fully understood.

**Fig 7:**
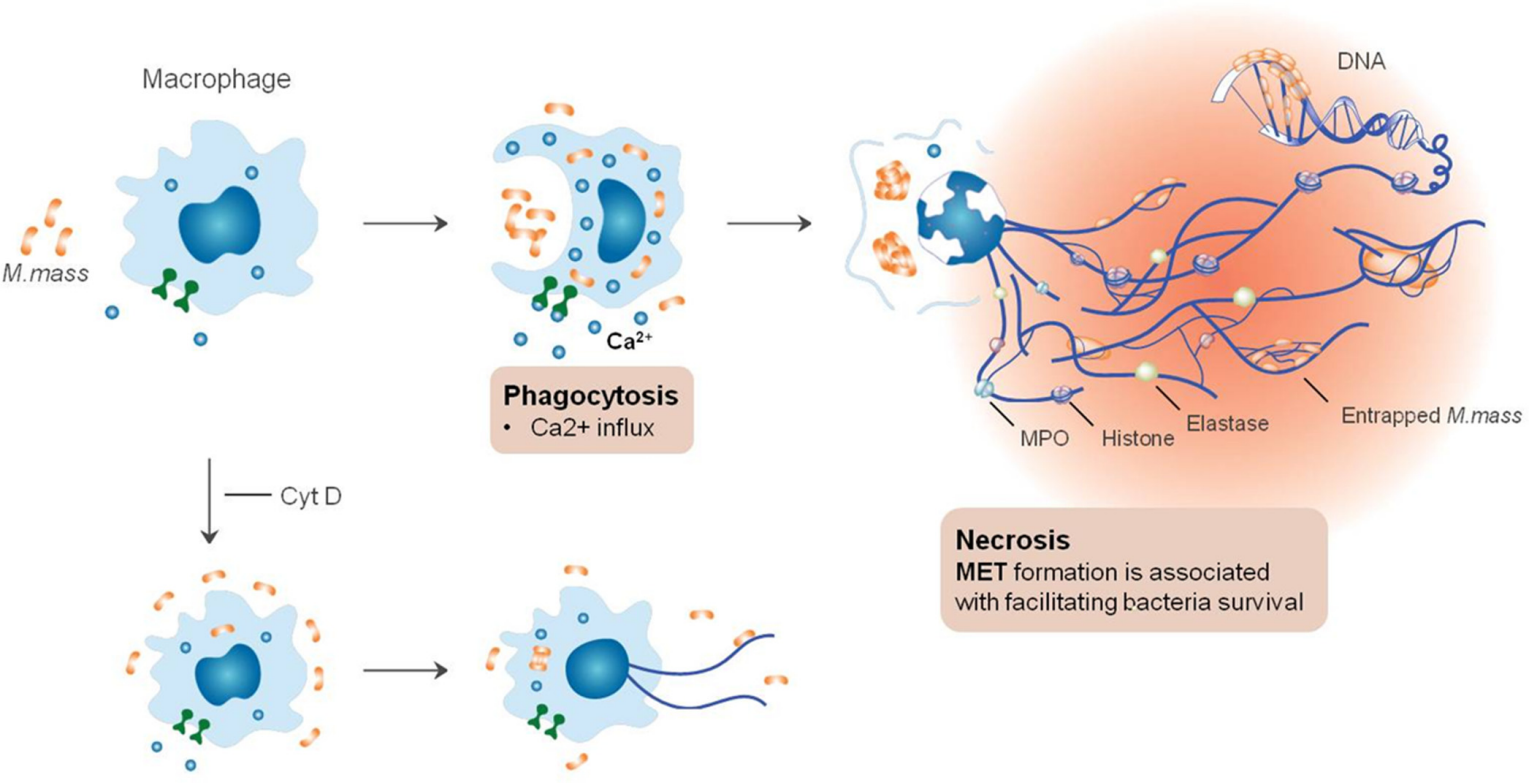
METs formation mediated by entry of *M*. *mass* into macrophage. (A) Phagocytosis of *M*. *mass* by macrophages induces an increase in the level of intracellular calcium. Then, accompanying necrosis, METs are released and prevent bacterial dissemination by entrapping *M*. *mass*. The METs comprise of histones and antimicrobial enzymes (elastase and MPO) but rather facilitate bacterial survival and growth without bactericidal effects. (B) Treatment with cytochalasin D (Cyt D) prevents phagocytosis of *M*. *mass* by macrophage, thereby resulting in decreased METs production.

In conclusion, our study revealed that rapid growing virulent mycobacteria *M*. *mass* infection causes differentiated THP-1 macrophages to release extracellular DNA traps (Fig 7). *M*. *mass*-induced METs have the distinct characteristics compared to NETs. Phagocytosis of *M*. *mass* by macrophages induces calcium influx and intracellular bacterial growth. And increase in intracellular bacterial number is followed by macrophage cell necrosis and subsequent METs release. In addition, instead of killing entrapped bacteria, METs may enhance intracellular growth of *M*. *mass*. This response may be closely related to sequelae of extracellular traps, such as chronic inflammation or autoimmune diseases [53, 54, 55]. However, METs retain the primary functions of entrapping *M*. *mass* and preventing bacterial spread. Furthermore, considering the importance of adaptive immune responses against *M*. *mass*, METs may enhance immune responses, priming microbes to be targeted by other immune cells and evoking subsequent immune responses including T cell recruitment and activation [56, 57, 58]. Thus, our data suggest that METs have complex functions on host interactions with mycobacteria, and more studies are required for elucidating their functions in the context of various immune cells *in vivo*.

## Supporting Information

S1 Fig METs formation induced by intracellular *M*. *mass* R. THP-1 macrophages were infected with *M*. *mass* R (5 MOI). For intracellular infection, infected cells were washed after 3hr of infection to eliminate extracellular bacteria. Then, the samples were cultured for 1 day and MET formation was quantified. MR, *M*. *mass* R; MR-in, intracellular *M*. *mass* R. ns, non-significant; **, p<0.01 by one-way ANOVA with Bonferroni’s post-test.
(TIF)

S2 Fig METs formation by single cells or aggregates of *M*. *mass*. THP-1 macrophages were infected with sonicated single cells or nonsonicated aggregates of *M*. *mass* R or CIP (5 MOI) for 1 day, and MET formation was quantified. MR, *M*. *mass* R single cells; MR-Ag, *M*. *mass* R aggregates; CIP, *M*. *mass* CIP single cells; CIP-Ag, *M*. *mass* CIP aggregates. ns, non-significant; *, p<0.01 by one-way ANOVA with Bonferroni’s post-test.
(TIF)

S3 Fig *M*. *mass*-induced METs formation in THP-1 macrophages stimulated by proinflammatory cytokines. A) Proinflammatory cytokines (TNF-α, IL-1β and IL-6) secreted from THP-1 macrophages infected with *M*. *mass* R or CIP. B) THP-1 macrophages were stimulated by IFN-γ and TNF-α (10ng/ml) for 24hr, and infected with *M*. *mass* R or CIP (5 MOI). The METs formation was examined at 1 day post infection. MR, *M*. *mass* R; CIP, *M*. *mass* CIP. ns, non-significant; *, p<0.05; **, p<0.01 by one-way ANOVA with Bonferroni’s post-test.
(TIF)

S4 Fig Intracellular ROS production in THP-1 macrophages infected with *M*. *mass* R. The quantification of ROS production in differentiated THP-1 macrophages stimulated with hydrogen peroxide or, *M*. *mass* with or without DPI by staining with ROS probe, H2DCF-DA. The mean fluorescence intensity (MFI) of the ROS probe was measured by flow cytometry analysis. The data are representative of three independent experiments. ns, non-significant by one-way ANOVA with Bonferroni’s post-test.
(TIF)

S5 Fig *M*. *mass* R-induced METs formation is not dependent of elastase activity. Differentiated THP-1 cells were untreated or pretreated with AAPV (0.1 or 1 μM) and infected with *M*. *mass* R (MOI 5) for 24 hrs. The MET formation was quantified as previously described in materials & methods. ns, non-significant; ***, p<0.001 compared to *M*. *mass* R-infected group by one-way ANOVA with Bonferroni’s post-test.
(TIF)

S6 Fig Cell death of THP-1 macrophages infected by *M*. *mass* R. Flow cytometry analysis of (A) uninfected or (B) infected THP-1 macrophages with *M*. *mass* (MOI 5) by staining with Annexin V (AV) and Propidium iodide (PI) at 1 day post infection. The numbers indicate the percentages of cells in each quadrant (lower left: AV-/PI-, live cells; lower right: AV+/ PI-, apoptotic cells; upper left: AV-/ PI+, necrotic cells; upper right: AV+/ PI +, late apoptotic or secondary necrotic cells). The data are representative of three independent experiments.
(TIF)

S7 Fig METs formation and cell lysis induced by *M*. *mass* CIP infection. (A) Cell lysis in THP-1 macrophages infected with *M*. *mass* CIP (MOI 10, 3, 1) at 2 days post infection. (B) MET formation in THP-1 macrophages infected with *M*. *mass* CIP for 2 days comparing with *M*. *mass* R-induced MET formation for 1 day. *, p<0.05; **, p<0.01; ***, p<0.001 by one-way ANOVA (A) or two-way ANOVA (B) with Bonferroni’s post-test.
(TIF)
