# Supplementary figures and images for: Mycobacterium massiliense Induces Macrophage Extracellular Traps with Facilitating Bacterial Growth
Source: PLoS One. 2016 May 18;11(5):e0155685. doi: 10.1371/journal.pone.0155685 (PMC4871462; doi:10.1371/journal.pone.0155685)

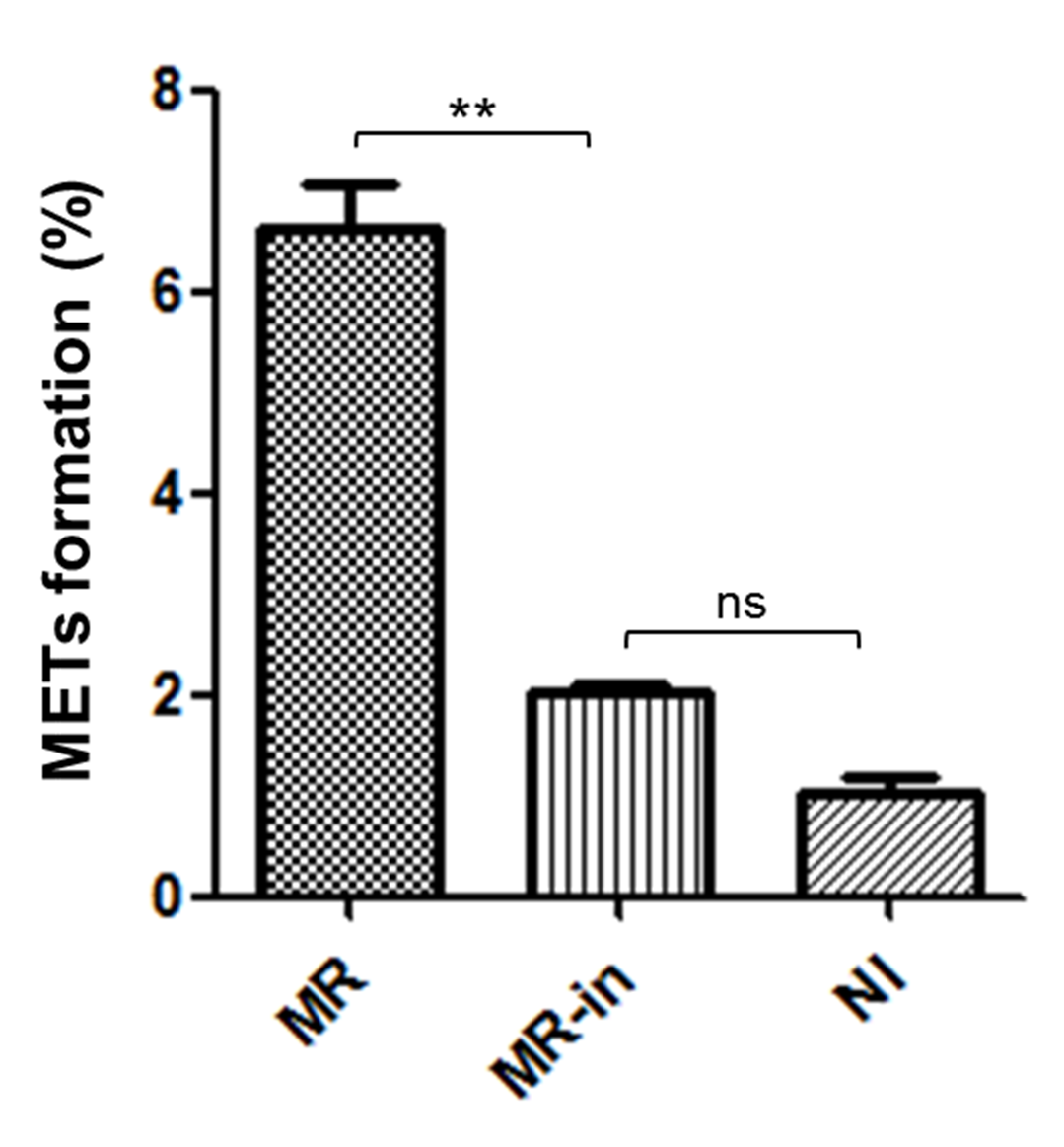

Supplement: S1 Fig — THP-1 macrophages were infected with M. mass R (5 MOI). For intracellular infection, infected cells were washed after 3hr of infection to eliminate extracellular bacteria. Then, the samples were cultured for 1 day and MET formation was quantified. MR, M. mass R; MR-in, intracellular M. mass R. ns, non-significant; **, p<0.01 by one-way ANOVA with Bonferroni’s post-test. (TIF) [file pone.0155685.s001.tif]

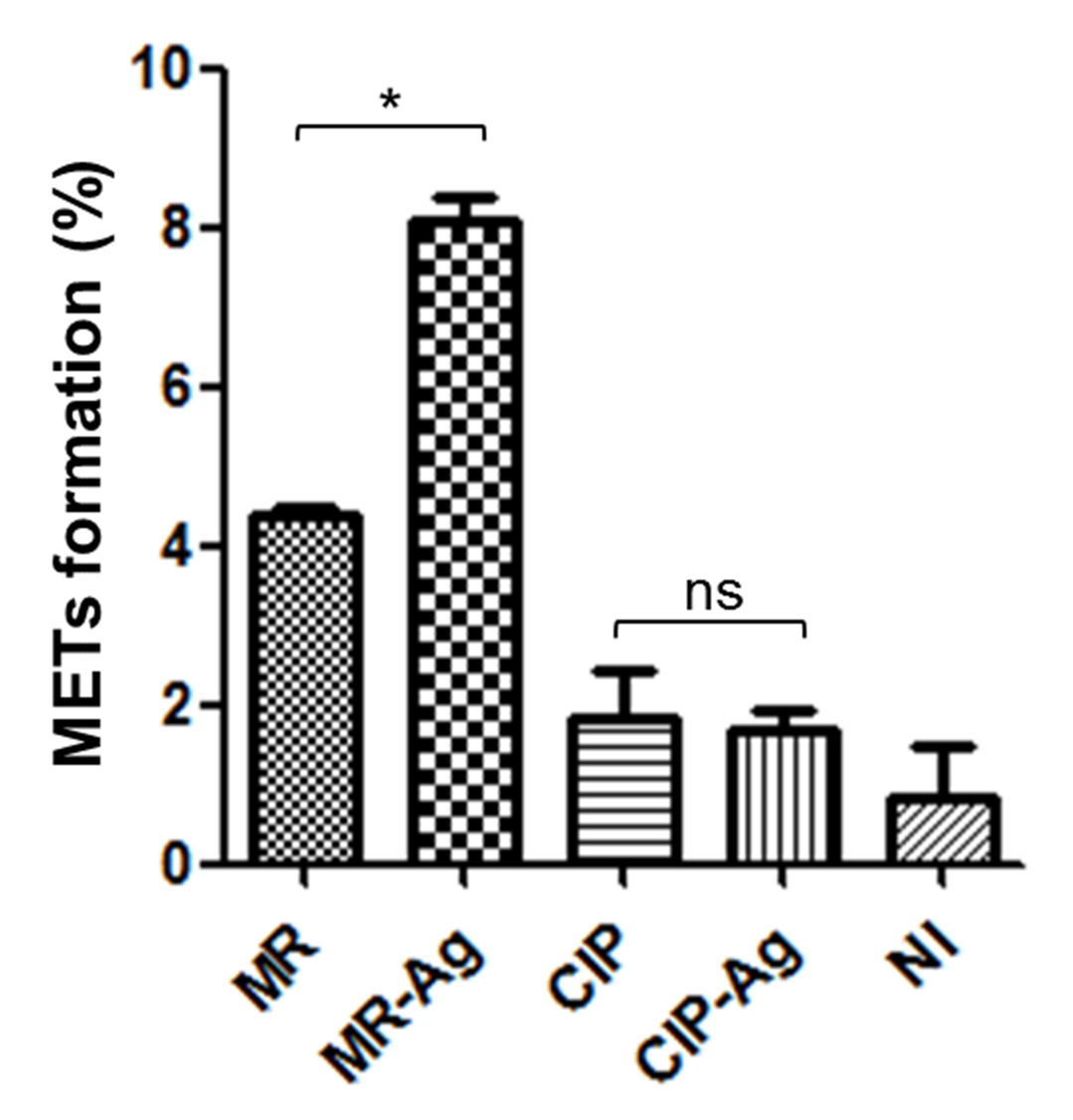

Supplement: S2 Fig — THP-1 macrophages were infected with sonicated single cells or nonsonicated aggregates of M. mass R or CIP (5 MOI) for 1 day, and MET formation was quantified. MR, M. mass R single cells; MR-Ag, M. mass R aggregates; CIP, M. mass CIP single cells; CIP-Ag, M. mass CIP aggregates. ns, non-significant; *, p<0.01 by one-way ANOVA with Bonferroni’s post-test. (TIF) [file pone.0155685.s002.tif]

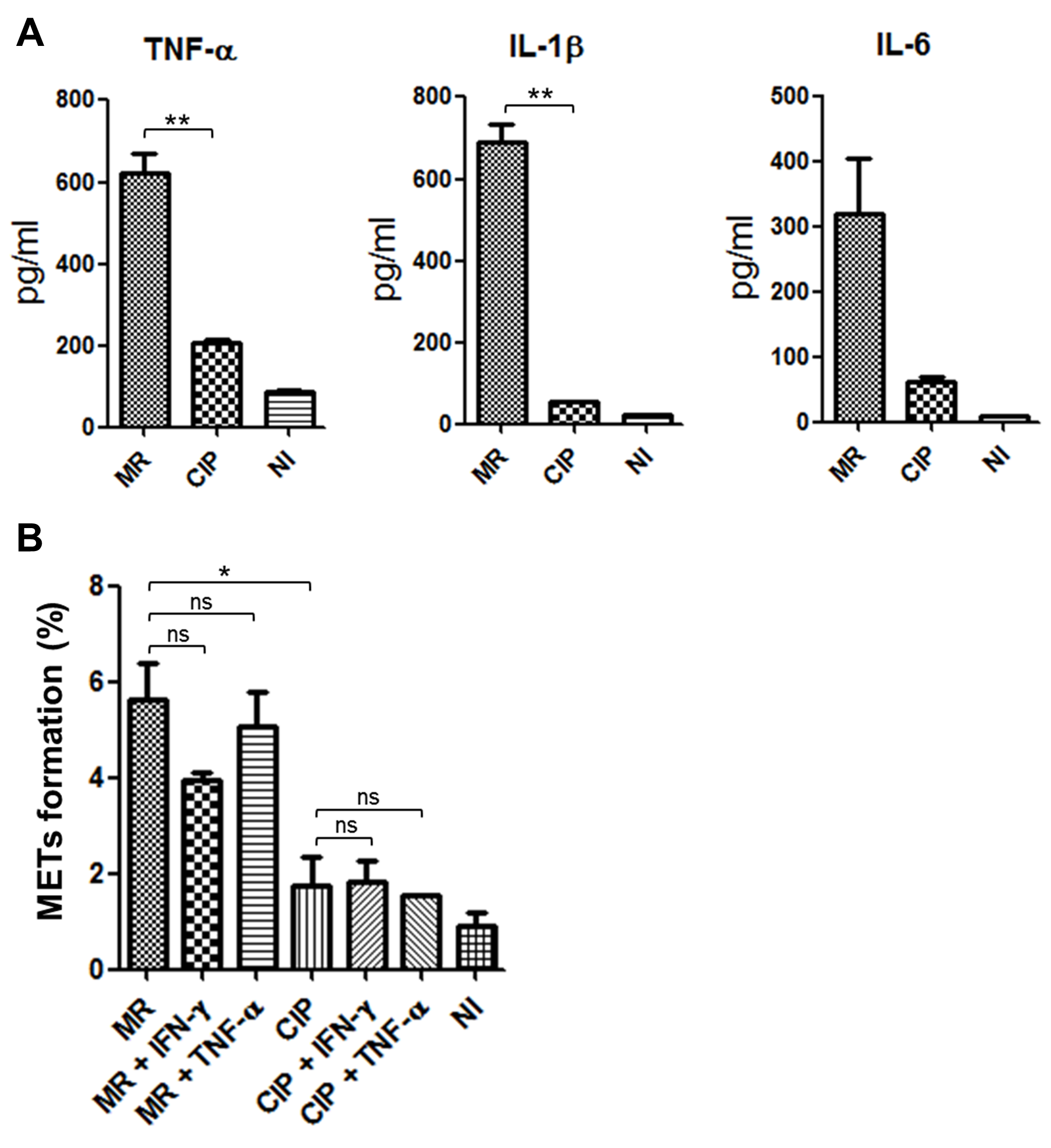

Supplement: S3 Fig — A) Proinflammatory cytokines (TNF-α, IL-1β and IL-6) secreted from THP-1 macrophages infected with M. mass R or CIP. B) THP-1 macrophages were stimulated by IFN-γ and TNF-α (10ng/ml) for 24hr, and infected with M. mass R or CIP (5 MOI). The METs formation was examined at 1 day post infection. MR, M. mass R; CIP, M. mass CIP. ns, non-significant; *, p<0.05; **, p<0.01 by one-way ANOVA with Bonferroni’s post-test. (TIF) [file pone.0155685.s003.tif]

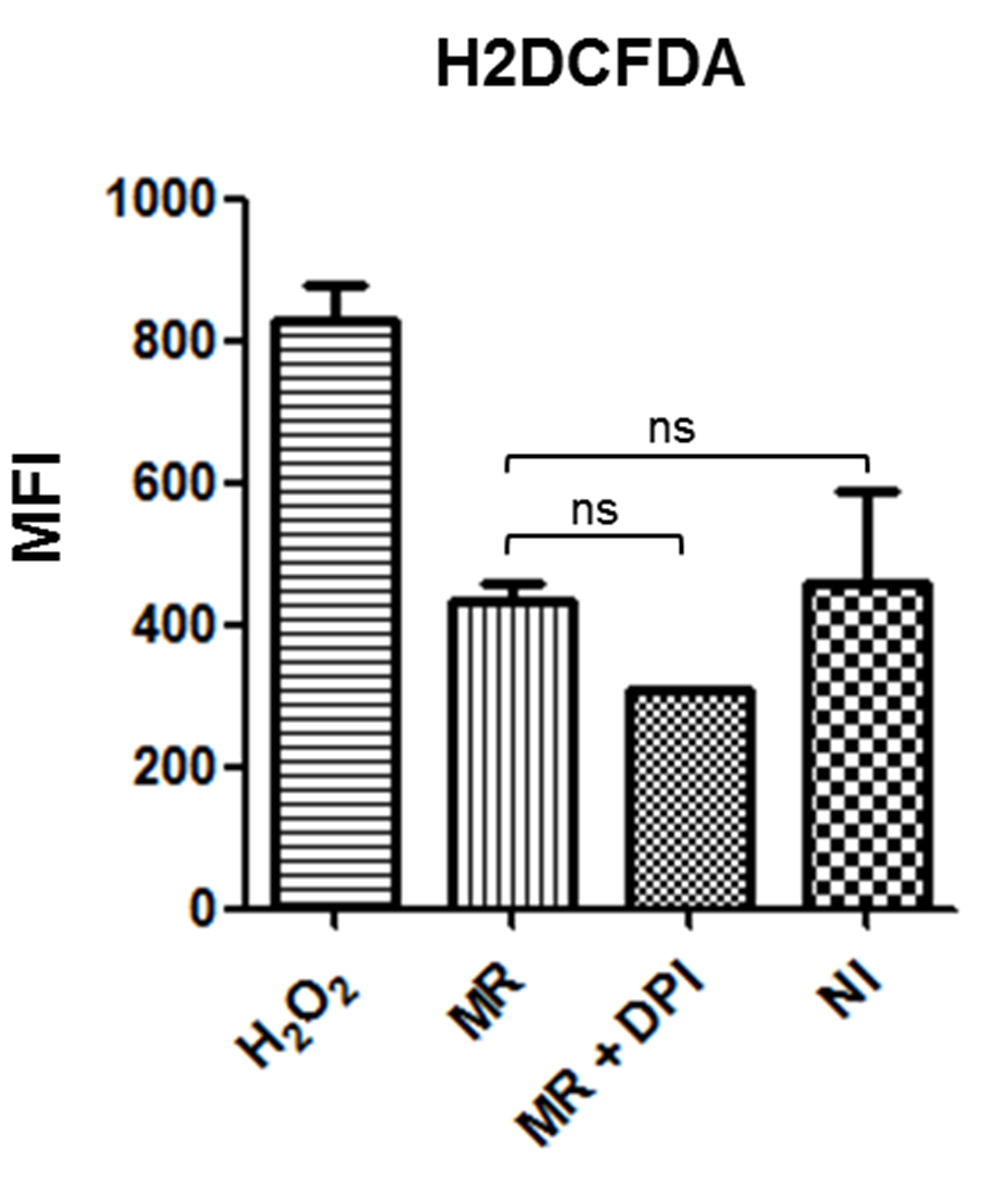

Supplement: S4 Fig — The quantification of ROS production in differentiated THP-1 macrophages stimulated with hydrogen peroxide or, M. mass with or without DPI by staining with ROS probe, H2DCF-DA. The mean fluorescence intensity (MFI) of the ROS probe was measured by flow cytometry analysis. The data are representative of three independent experiments. ns, non-significant by one-way ANOVA with Bonferroni’s post-test. (TIF) [file pone.0155685.s004.tif]

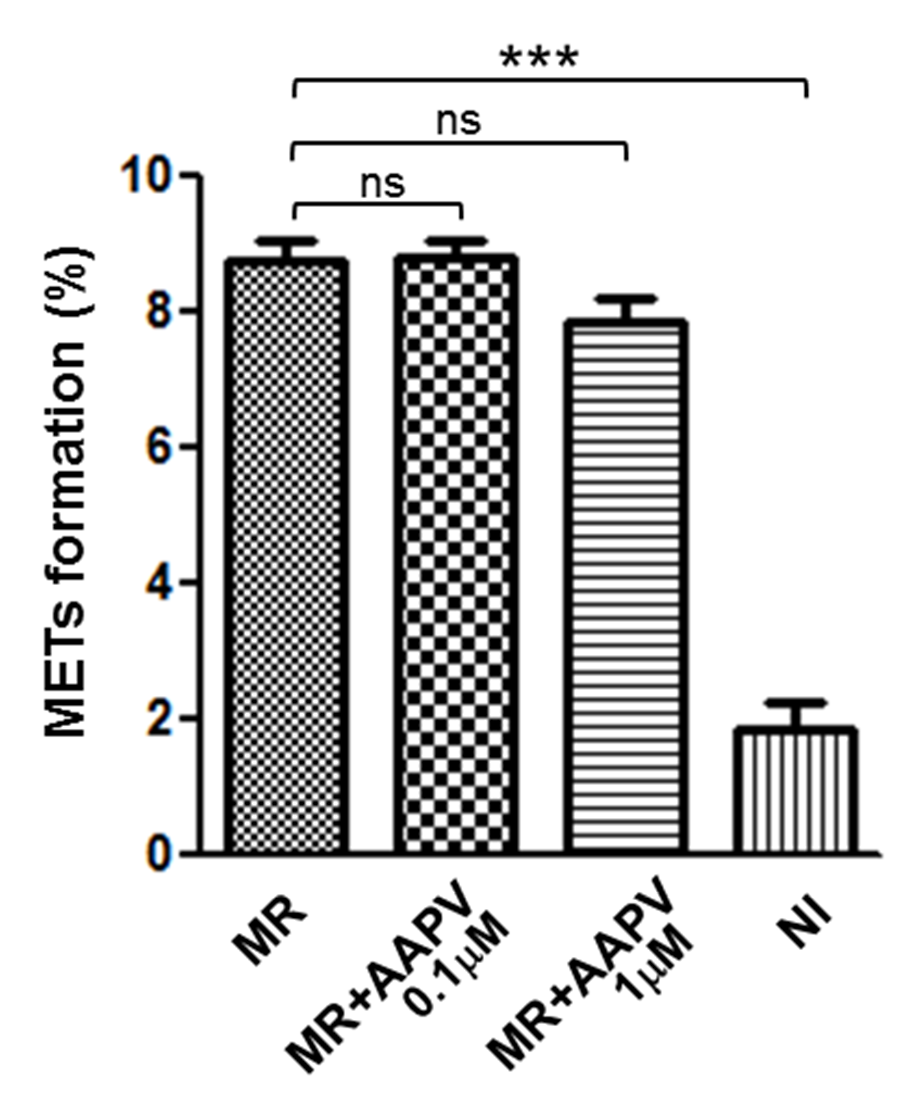

Supplement: S5 Fig — Differentiated THP-1 cells were untreated or pretreated with AAPV (0.1 or 1 μM) and infected with M. mass R (MOI 5) for 24 hrs. The MET formation was quantified as previously described in materials & methods. ns, non-significant; ***, p<0.001 compared to M. mass R-infected group by one-way ANOVA with Bonferroni’s post-test. (TIF) [file pone.0155685.s005.tif]

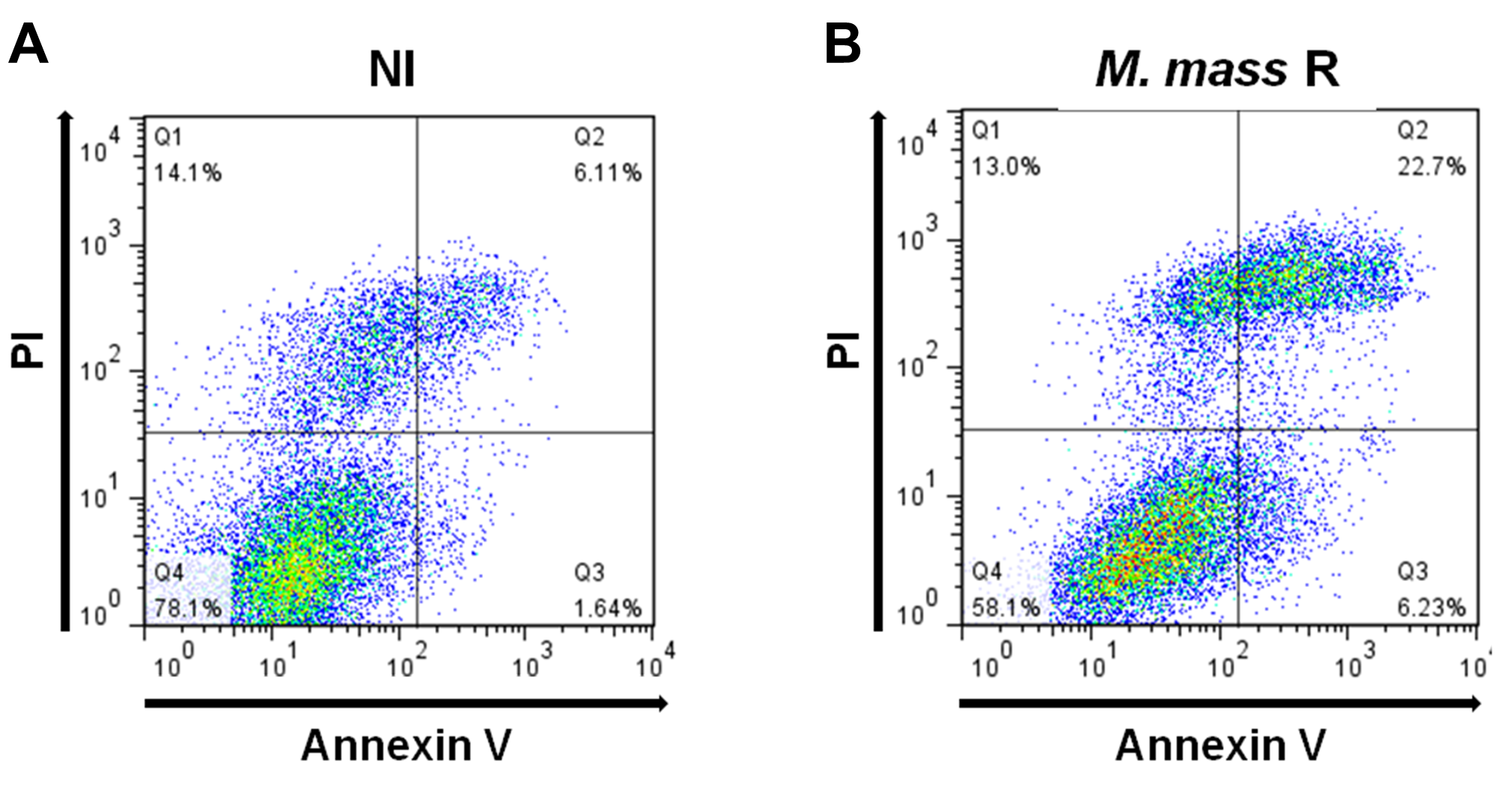

Supplement: S6 Fig — Flow cytometry analysis of (A) uninfected or (B) infected THP-1 macrophages with M. mass (MOI 5) by staining with Annexin V (AV) and Propidium iodide (PI) at 1 day post infection. The numbers indicate the percentages of cells in each quadrant (lower left: AV-/PI-, live cells; lower right: AV+/ PI-, apoptotic cells; upper left: AV-/ PI+, necrotic cells; upper right: AV+/ PI +, late apoptotic or secondary necrotic cells). The data are representative of three independent experiments. (TIF) [file pone.0155685.s006.tif]

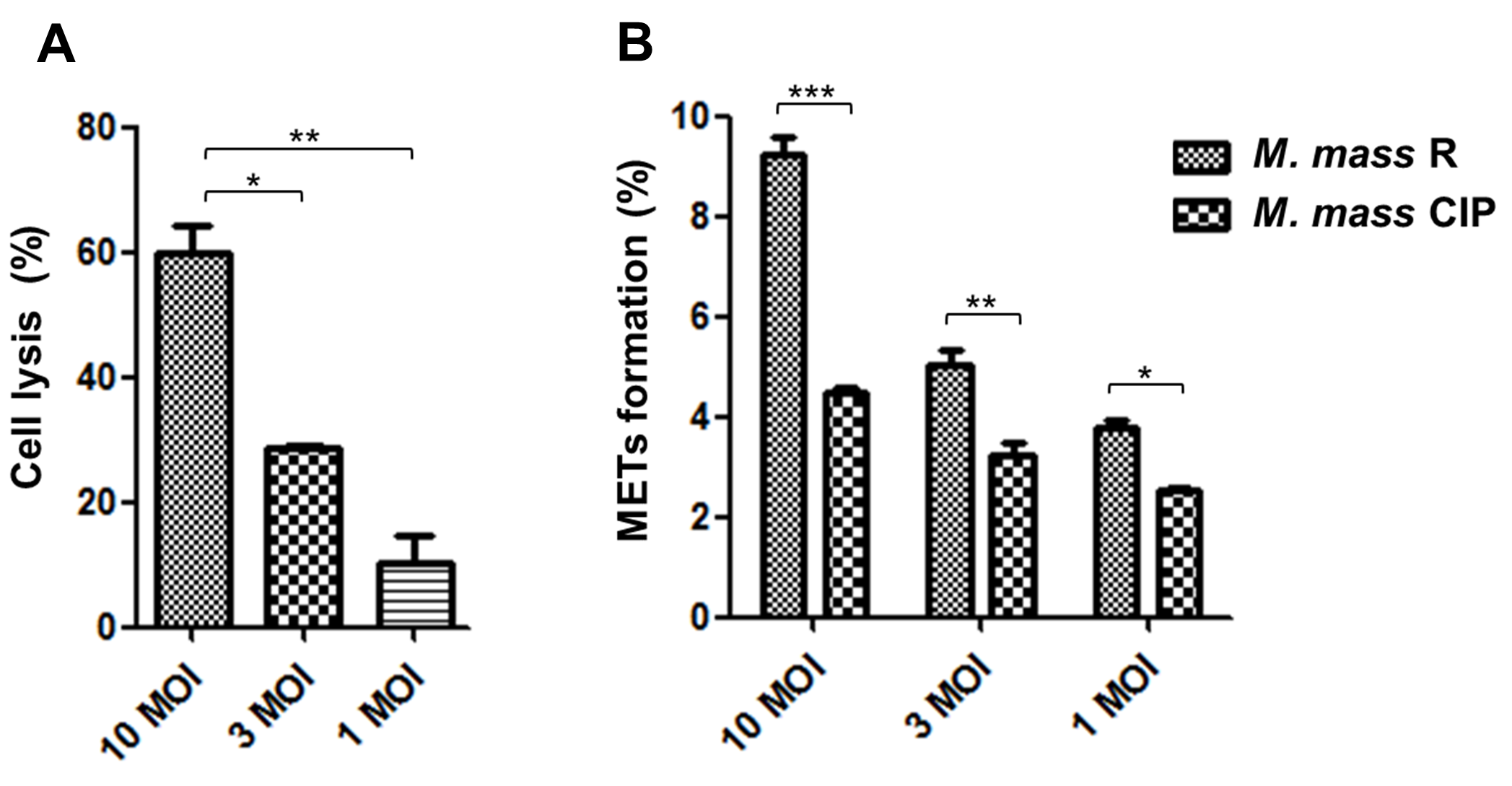

Supplement: S7 Fig — (A) Cell lysis in THP-1 macrophages infected with M. mass CIP (MOI 10, 3, 1) at 2 days post infection. (B) MET formation in THP-1 macrophages infected with M. mass CIP for 2 days comparing with M. mass R-induced MET formation for 1 day. *, p<0.05; **, p<0.01; ***, p<0.001 by one-way ANOVA (A) or two-way ANOVA (B) with Bonferroni’s post-test. (TIF) [file pone.0155685.s007.tif]
